# Regulation of Genes Involved in Carnitine Homeostasis by PPAR****α**** across Different Species (Rat, Mouse, Pig, Cattle, Chicken, and Human)

**DOI:** 10.1155/2012/868317

**Published:** 2012-10-23

**Authors:** Robert Ringseis, Gaiping Wen, Klaus Eder

**Affiliations:** Institute of Animal Nutrition and Nutrition Physiology, Justus-Liebig-University Giessen, Heinrich-Buff-Ring 26-32, 35390 Giessen, Germany

## Abstract

Recent studies in rodents convincingly demonstrated that PPAR**α** is a key regulator of genes involved in carnitine homeostasis, which serves as a reasonable explanation for the phenomenon that energy deprivation and fibrate treatment, both of which cause activation of hepatic PPAR**α**, causes a strong increase of hepatic carnitine concentration in rats. The present paper aimed to comprehensively analyse available data from genetic and animal studies with mice, rats, pigs, cows, and laying hens and from human studies in order to compare the regulation of genes involved in carnitine homeostasis by PPAR**α** across different species. Overall, our comparative analysis indicates that the role of PPAR**α** as a regulator of carnitine homeostasis is well conserved across different species. However, despite demonstrating a well-conserved role of PPAR**α** as a key regulator of carnitine homeostasis in general, our comprehensive analysis shows that this assumption particularly applies to the regulation by PPAR**α** of carnitine uptake which is obviously highly conserved across species, whereas regulation by PPAR**α** of carnitine biosynthesis appears less well conserved across species.

## 1. Introduction

Peroxisome proliferator-activated receptor *α* (PPAR*α*) is considered a master transcriptional regulator of lipid metabolism and energy homeostasis [1], because typical genes regulated by PPAR*α* are involved in all aspects of fatty acid catabolism (cellular fatty acid uptake, activation of fatty acids, intracellular fatty acid transport, import of fatty acids into the mitochondria, and mitochondrial and peroxisomal fatty acid *β*-oxidation), ketogenesis, as well as gluconeogenesis [2]. PPAR*α*-dependent gene transcription is initiated when a ligand, for example, fatty acids which are released from white adipose tissue during energy deprivation and taken up into tissues during this state, or exogenous ligands such as fibrates (WY-14,643, clofibrate, fenofibrate, bezafibrate, and gemfibrozil), binds to the ligand-binding domain of this transcription factor. Mechanistic details of gene regulation by PPAR*α* and tissue distribution of PPAR*α* has been extensively described in the literature, wherefore the reader is referred to the literature with regard to this [3]. Interestingly, earlier studies repeatedly reported that energy deprivation or treatment of rats with fibrates causes a marked, up to 5-fold elevation of the hepatic concentration of carnitine [4–7]. The molecular mechanisms underlying this phenomenon, however, have not been resolved from these studies. It was not until about twenty years later that activation of hepatic PPAR*α*, which is common to energy deprivation and fibrate treatment, was shown to cause an increase in the expression of genes involved in carnitine uptake and biosynthesis in liver cells [8] serving as a reasonable explanation for the abovementioned phenomenon. In subsequent studies it was shown that elevation of hepatic carnitine concentration in response to fasting, or fibrates occurs only in wild-type mice but not in transgenic mice lacking a functional PPAR*α* protein strengthening the assumption that PPAR*α* is a critical regulator of carnitine homeostasis [9, 10]. Using more sophisticated molecular biological techniques it could be convincingly demonstrated that the mouse genes encoding the carnitine transporter novel organic cation transporter 2 (OCTN2/SLC22A5) and two enzymes of the carnitine biosynthetic pathway, *γ*-butyrobetaine dioxygenase (BBOX1) and 4-trimethylaminobutyraldehyde dehydrogenase (ALDH9A1), are direct PPAR*α* target genes as evidenced by the identification of functional PPRE within the regulatory region of the respective genes [11–13].

It is well established that PPAR*α* activators exert distinct species-specific actions [14–17]. In rodents, like mice and rats, administration of PPAR*α* activators leads to a marked peroxisomal enzyme induction, peroxisome proliferation, and even hepatocarcinogenesis [18, 19]. In contrast, PPAR*α* activators cannot induce peroxisome proliferation and hepatocarcinogenesis and the induction of peroxisomal metabolism pathways is much less pronounced in human hepatocytes and livers from nonhuman primates [17]. This distinct response of the peroxisomes to PPAR*α* activators is responsible for the classification of different species into proliferating (mice, rats) and nonproliferating ones (humans, monkeys, guinea pigs). Several factors are considered to account for the marked difference in the response to PPAR*α* activators between different species: expression level of PPAR*α*, degree of conservation and functionality of the PPRE in the regulatory region of target genes, and lack or overexpression of transcriptional coregulators [17]. Apart from these marked differential effects of PPAR*α* activators on peroxisome proliferation between proliferating and nonproliferating species, a comparative analysis of gene regulation by PPAR*α* between mouse and human revealed that at least the role of PPAR*α* as a master regulator of hepatic lipid catabolism is well conserved [20]. It may be therefore expected that regulation of carnitine homeostasis, which is intrinsically linked to fatty acid catabolism because the transport of fatty acids from the cytosol into the mitochondrial matrix for subsequent fatty acid *β*-oxidation is carnitine-dependent, is also a well conserved function of PPAR*α*. However, despite its well conserved role as an important regulator of lipid catabolism in general, the specific genes under control of PPAR*α* within each lipid metabolic pathway were shown to differ at least between humans and mice [20]. Thus, whether PPAR*α* can be considered as a critical regulator of genes involved in carnitine homeostasis across different species requires thorough analysis of the effect of PPAR*α* activation on carnitine homeostasis in each individual species and cannot be predicted for one species by simply transferring observations obtained in mice or rats. In light of the abovementioned species specificities with regard to the response to PPAR*α* activation the present paper aims to (1) briefly describe current knowledge about the genes involved in the regulation of carnitine homeostasis and (2) to comprehensively analyse available data from genetic and animal studies with mice, rats, pigs, cows, and laying hens and from human studies in order to compare the regulation of genes involved in carnitine homeostasis by PPAR*α* across different species. 

## 2. Regulation of Carnitine Homeostasis

Carnitine is a water soluble quaternary amine (3-hydroxy-4-N,N,N-trimethylaminobutyric acid) which is essential for normal function of all tissues. The primary function of carnitine is to facilitate the translocation of activated long-chain fatty acids from the cytosol into the mitochondrial matrix, a process called carnitine shuttle, for subsequent fatty acid *β*-oxidation. In mammals, carnitine is considered a conditionally essential nutrient because it is synthesized by the organism but most is taken up from the diet [21]. Food of animal origin, such as meat and dairy products, containing high carnitine levels, makes the greatest contribution to total carnitine uptake. In contrast, the intake of food of plant origin is negligible for dietary carnitine uptake due to its very low carnitine levels [22]. Thus, dietary uptake of carnitine in strict vegetarians is very low and has been estimated to be less than 0.02 mg per kg body weight and day [23], whereas dietary carnitine uptake through an omnivorous diet provides approximately 0.3–1.9 mg carnitine per kg body weight and day. Nonetheless, plasma carnitine levels in vegetarians are only 15–30% lower than those in nonvegetarians, being yet within the normal physiological range (25–50 *μ*mol/L), because vegetarians have a more efficient renal reabsorption of carnitine (urinary total carnitine excretion was 55% less in vegetarians than in nonvegetarians [24]) and a greater rate of endogenous carnitine biosynthesis [25, 26]. In healthy vegetarians, carnitine deficiency (plasma carnitine concentration < 25 *μ*mol/L [26]) may develop only if certain micronutrients, such as vitamin C, vitamin B_6_, and iron, which are required as co-factors for carnitine biosynthesis are not provided from the diet in sufficient amounts. The tubular reabsorption of carnitine in the kidney, where >95% of filtered free carnitine is reabsorbed when plasma free carnitine concentration is within the normal range, is of great importance for maintaining normal plasma carnitine levels. This is evidenced by the fact that patients with inborn or acquired defects in this tubular carnitine reabsorption process develop primary systemic carnitine deficiency with markedly reduced serum carnitine levels (0–5 *μ*mol/L) because most of the filtered carnitine is lost in the urine [27]. If plasma carnitine concentration exceeds the normal range (supraphysiologic levels) due to the uptake of high dosages of carnitine (e.g., oral or i.v. supplementation), the excess carnitine is rapidly eliminated due to saturation of the tubular reabsorption mechanism [26, 28]. This explains the fact that the ability to maintain supra-physiologic plasma carnitine concentrations is limited [29, 30]. The skeletal muscle contains the majority of the total body carnitine [31], and, like the myocardium, is dependent on the active uptake of carnitine from plasma against a strong concentration gradient (from 25–50 *μ*mol/L in plasma to about 4000 *μ*mol/L in skeletal muscle) [32]. Due to this large endogenous carnitine pool, a single intravenous dose of carnitine or short-term oral supplementation with carnitine at high doses (4–6 g/day) has little or no impact on the muscle carnitine content [33–35]. 

## 3. Genes Encoding Proteins Involved in Carnitine Homeostasis

### 3.1. Carnitine Biosynthesis

The carnitine biosynthesis pathway consists of a cascade of four distinct enzymatic reactions through which 6-N-trimethyllysine (TML), which is the substrate for carnitine biosynthesis, is converted stepwise into carnitine. TML is the product of lysosomal and proteasomal degradation of proteins containing N-methylated lysines, such as calmodulin, myosin, actin, and histones [21]. In the first enzymatic step, TML is hydroxylated by the enzyme TML dioxygenase (encoded by TMLHE) to yield 3-hydroxy-TML (HTML). Subsequently, the second enzyme, called HTML aldolase (encoded by HTMLA), catalyzes an aldolytic cleavage of HTML, which results in the formation of 4-trimethylaminobutyraldehyde (TMABA). The third enzyme, called TMABA dehydrogenase (encoded by ALDH9A1), catalyzes the dehydrogenation of TMABA to 4-N-trimethylaminobutyrate or *γ*-butyrobetaine (BB). In the final biosynthetic step, BB is hydroxylated by BB dioxygenase (encoded by BBOX1) to form carnitine [21]. In all mammals, a significant BBD activity is found in the liver [36], and in some species such as in humans, pigs, cats, cows, hamsters, rabbits, or Rhesus monkeys also in the kidney [36, 37]. Other tissues than liver and kidney have either no or only a very low activity of BBD [36, 37], and are therefore highly dependent on active carnitine uptake from blood. The BB, which is formed in extrahepatic tissues, is excreted and transported via the circulation to the liver, where it is converted into carnitine [36].

### 3.2. Carnitine Uptake

Tissues which are incapable of providing carnitine via endogenous biosynthesis, such as skeletal muscle and myocardium, are highly dependent on the uptake of carnitine from the circulation. This transport across the plasma membrane against a high concentration gradient (in skeletal muscle > 100-fold) is mediated by the novel organic cation transporters (OCTNs) which belong to the solute carrier 22A family [38, 39]. The OCTN2 isoform, which is sodium-dependent and high-affinity, is considered the physiologically most important one due to its wide tissue expression [40, 41]. This transporter represents the molecular basis for the tubular reabsorption process of carnitine in the kidney and is therefore fundamental for maintaining normal carnitine levels in serum. As mentioned above, defects in the renal reabsorption process of carnitine due to a mutation in the OCTN2 gene are causative for severe carnitine deficiency in such patients [42]. In the small intestine, OCTN2 also plays a key role for the absorption of carnitine from the diet [43]. This is based on the observation that in mice with a genetic defect in the OCTN2 gene oral bioavailability of carnitine was reduced by approximately 50% [44].

The OCTN1 isoform is considered to contribute less to carnitine transport than OCTN2 due to its low carnitine transport activity. OCTN1 is localized in the mitochondrial membrane in close proximity to CPT I, the rate-limiting enzyme for carnitine-dependent fatty acid oxidation. Due to this localization, OCTN1 has been proposed to operate on the mitochondrial influx and efflux of carnitine and acylcarnitine esters indicating that OCTN1 is mainly involved in maintaining intracellular carnitine homeostasis [45]. Another OCTN isoform, namely, OCTN3, has been suggested to play a role for carnitine uptake into testis and to also mediate renal reabsorption of carnitine [41].

## 4. Evidence for a Role of PPAR***α*** in Regulating Genes Involved in Carnitine Homeostasis in Different Species

### 4.1. Rat

Based on earlier reports that energy deprivation or treatment with fibrates, both of which induce activation of hepatic PPAR*α*, causes a marked elevation of the hepatic carnitine concentration [4–7], we have recently tested the hypothesis that PPAR*α* activation is responsible for this phenomenon. Indeed, we demonstrated for the first time that PPAR*α* activators strongly increase transcript levels of OCTN2 in rat liver and cultivated rat hepatocytes [8]. Moreover, we found that the increase in OCTN2 mRNA abundance in response to treatment with PPAR*α* activators was accompanied by an elevation of the carnitine concentration in rat liver and cultivated rat hepatocytes [8]. These findings provided the first evidence that PPAR*α* plays a role in regulating carnitine homeostasis through stimulating OCTN2-mediated carnitine uptake from blood into the liver. In subsequent studies with rats, we found that treatment with PPAR*α* activators increases also OCTN2 transcript levels in small intestine [46, 47], and improves intestinal carnitine absorption [47]. Thus, these observations confirmed our assumption that PPAR*α* is an important regulator of carnitine uptake and that upregulation of OCTN2 in small intestine may contribute to the elevation of hepatic carnitine concentration in response to PPAR*α* activators through increasing carnitine availability from the diet. A further study in rats revealed that energy deprivation, which is a physiologic state of PPAR*α* activation, also results in increases of OCTN2 transcript levels and carnitine concentration in the liver [48]. Since administration of oxidized fats causes a strong activation of PPAR*α* in rats [49–51] due to the high content of hydroxylated fatty acids and cyclic fatty acid monomers, both of which are ligands of PPAR*α*, we also investigated whether feeding of oxidized fats causes similar effects on carnitine homeostasis as energy deprivation and fibrate treatment [52]. Indeed, we observed that administration of oxidized fat for 6 d causes an elevation of OCTN2 transcript levels in liver and small intestine and increases hepatic carnitine concentration of rats indicating that carnitine homeostasis is regulated also by nutritive PPAR*α* activators.

Since the results from these experiments suggested that OCTN2 might be a direct target gene of PPAR*α*, we performed in silico analysis of the rat OCTN2 promoter which revealed several putative PPRE upstream of the transcription start site [46]. Using reporter gene and gel mobility shift assays, Maeda et al. [53] recently identified one functional PPRE in the rat OCTN2 promoter confirming our assumption that the rat OCTN2 gene is a direct PPAR*α* target gene. However, in comparison to the marked induction of OCTN2 mRNA by fibrates and fasting [8, 46, 48] the weak stimulation of rat OCTN2 promoter activity reported from Maeda et al. [53] suggested that a more potent PPRE, located in other regulatory regions than the proximal promoter, might be responsible for OCTN2 upregulation in response to PPAR*α* activation.

Although previous studies in rats indicated that the clofibrate-induced increase in hepatic carnitine concentration is due to an increase in the rate of hepatic carnitine synthesis [6, 7], results from analysis of gene expression of enzymes of the carnitine biosynthesis pathway in rats do not point towards a role for PPAR*α* in regulating genes involved in carnitine biosynthesis in rats. All of the abovementioned studies in rats did not show any increase in the transcript levels of ALDH9A1 and BBOX1 in the liver in response to fibrate treatment, fasting, or administration of oxidized fat. This indicates that at least the rat genes encoding ALDH9A1 and BBOX1 are not transcriptionally regulated by PPAR*α*, despite the fact that several conserved PPRE were identified in the proximal promoter of the rat BBOX1 gene using NUBIScan software [9]. However, studies in rats demonstrated that both clofibrate and fasting increase the concentration of the carnitine precursor TML in the liver [46, 48, 54]. Since carnitine biosynthesis starts with the enzymatic conversion of TML, the availability of TML has been considered to be rate limiting for carnitine biosynthesis [55]. In fact, TML is subsequently converted into BB, which itself is rapidly further converted into carnitine due to the large capacity of the liver to convert BB into carnitine [56]. Thus, it is possible that carnitine biosynthesis is stimulated by PPAR*α* activation, an effect that is not mediated by increasing expression of genes encoding enzymes of the carnitine biosynthesis pathway but rather by stimulating lysosomal and proteasomal degradation of proteins which leads to the release of TML [57, 58]. The observation that both clofibrate and fasting stimulate proteolysis [59] is supportive for this assumption.

### 4.2. Mouse

According to convincing data from studies with rats that PPAR*α* plays a role in the regulation of carnitine homeostasis, studies with PPAR*α* knockout and corresponding wild-type mice have been conducted [9, 10]. van Vlies et al. [9] were the first demonstrating that PPAR*α* regulates gene expression of OCTN2 in the liver of mice as evidenced by the observation that upregulation of OCTN2 in response to fasting or WY-14,643 treatment occurs only in wild-type but not in PPAR*α* knockout mice. Using the same mice genotypes, Koch et al. [10] largely confirmed these findings from van Vlies et al. [9] but additionally demonstrated that PPAR*α* activators cause OCTN2 upregulation also in kidney and small intestine. Studies from both groups showed that the elevation of hepatic carnitine concentration in response to PPAR*α* activation occurs only in wild-type mice [9, 10], which provided further evidence that PPAR*α* is a critical player for regulating carnitine homeostasis. Noteworthy, these studies revealed also upregulation of genes encoding the carnitine biosynthetic enzymes ALDH9A1 and BBOX1 in the liver of wild-type but not PPAR*α* knockout mice indicating that genes involved in carnitine biosynthesis are regulated by PPAR*α* in mice, which is in contrast to the rat. 

Further indication for the PPAR*α* dependency of regulation of the mouse genes encoding OCTN2, ALDH9A1, and BBOX1 is provided by the observation that hepatic mRNA, and protein levels of OCTN2, ALDH9A1, and BBOX1 are decreased in obese mice compared to lean mice [60], because high fat diet-induced obesity was reported to disrupt hepatic PPAR*α* function and to impair PPAR*α* dependent gene transcription [61, 62]. Noteworthy, this study showed that the reduced hepatic expression levels of OCTN2, ALDH9A1, and BBOX1 were partially restored to expression levels of lean mice in a subgroup of the obese mice which were regularly exercised on a motorized treadmill (35 min, 5 x/wk, 10 wk). Since endurance exercise causes activation of PPAR*α*, these data suggest that endurance exercise was able to restore at least in part the obesity-induced disruption of PPAR*α* function and thereby contributed to the elevated gene expression of OCTN2, ALDH9A1, and BBOX1.

Besides direct transcriptional regulation of genes involved in carnitine homeostasis by PPAR*α*, evidence has been provided that PPAR*α* might influence the availability of requisite biosynthetic precursors—through the abovementioned stimulatory effect of PPAR*α* activation on proteolysis—and enzymatic cofactors required for carnitine synthesis. In this context a study from Makowski et al. [63] is worth mentioning which reported that PPAR*α* knockout mice display markedly lower levels of methionine, which serves as a methyl donor during posttranslational assembly of methylated proteins, and *α*-ketoglutarate, which is a cofactor of TMLHE and BBOX1, in plasma and tissues, respectively, than wild-type mice.

Recent molecular biological studies by our own group revealed that the mouse genes encoding OCTN2, BBOX1, and ALDH9A1 are direct PPAR*α* target genes [11–13], which is in line with the abovementioned observations from studies with PPAR*α* knockout mice [9, 10]. Direct regulation of these genes by PPAR*α* was evidenced by the identification of one functional PPRE each in the regulatory region of these genes. The functional PPREs were shown to be located in either the proximal promoter (BBOX1 and ALDH9A1; [12, 13]) or the first intron (OCTN2; [11]). Taken together, these findings confirm that PPAR*α* plays a key role in the regulation of carnitine homeostasis in the mouse by controlling genes involved in carnitine synthesis and carnitine uptake.

### 4.3. Pig

The abovementioned observations in rodents cannot be directly applied for humans, because of marked differences in the response to PPAR*α* activators between rodents and humans [17, 18]. In contrast to rodents, pigs have a low expression of PPAR*α* in the liver and the response to PPAR*α* activators (induction of peroxisomal metabolism pathways, peroxisome proliferation) is very weak, wherefore pigs like humans and nonhuman primates belong to the nonproliferating species. A recent study from our group showed that PPAR*α* mRNA levels in the liver are comparable between pig and human [64], which suggests that the pig is a suitable model for humans to study the effects of PPAR*α* activation. Activation of PPAR*α* in liver and other tissues of pigs have been already demonstrated in response to clofibrate, oxidized fat as well as fasting [65–67]. In order to study whether carnitine homeostasis is regulated by PPAR*α* also in pigs we performed two experiments in which pigs were either treated with clofibrate or fasted for 24 h. Treatment with clofibrate caused an upregulation of OCTN2 in liver, skeletal muscle, and small intestine, and increased carnitine concentrations in liver and skeletal muscle [68]. Upregulation of OCTN2 in the liver and elevated carnitine concentrations in liver and kidney were also found in pigs which were fasted for a period of 24 h [67]. In the latter study, fasting was also shown to increase BBOX1 mRNA level and BBOX1 activity in liver and kidney [67]. Thus, these observations from studies with pigs indicate that carnitine homeostasis in pigs is also regulated by PPAR*α*, even though the extent of upregulation of OCTN2 and BBOX1 is lower in pigs than in rodents. The latter may be explained by the lower tissue expression level of PPAR*α* in pigs than in rodents but also by species differences in the availability of transcriptional coregulators. In this context it is worth mentioning that a large number of PPAR related coregulators, such as CBP/p300, SRC-1-3, PGC-1*α*, PGC-1*β*, PRIP, PRIC285, CARM1, and PIMT, have been described to influence PPAR target gene transcription and that their relative availability in a given tissue is at least partially responsible for the tissue specific expression of target genes and the responsiveness of PPAR isotypes to specific ligands [69]. 

### 4.4. Cattle

In contrast to the large body of literature with regard to the regulation of carnitine homeostasis by PPAR*α* in rodents, only limited information is available on the regulation of PPAR*α* activity and its role for carnitine homeostasis in cattle liver. Apart from demonstrating that PPAR*α* is functional in cattle liver [70] and long-chain fatty acids are able to activate PPAR*α* in bovine cells [71, 72], it was shown that the negative energy balance occurring in early lactating dairy cows is associated with an upregulation of several established PPAR*α* target genes in nonruminants in the liver being indicative of PPAR*α* activation during early lactation [73–76]. Based on previous observations that hepatic carnitine concentration in dairy cows is increasing during the transition from late pregnancy to early lactation [77, 78], we have recently investigated whether hepatic genes of carnitine synthesis and uptake of carnitine are upregulated during early lactation in dairy cows [79]. As expected and in accordance with results from a recent study [73], our study showed that the negative energy balance occurring at early lactation was associated with elevated plasma levels of free fatty acids and increased transcript levels of established PPAR*α* target genes in nonruminants [79], which is indicative of activation of hepatic PPAR*α* in early lactating cows. In line with our hypothesis, our study showed that the transition from late pregnancy (3 wk prepartum) to early lactation leads to an upregulation of various genes involved in carnitine synthesis (ALDH9A1, TMLHE, BBOX1) and carnitine uptake (OCTN2) in the liver of cows at 1 wk postpartum [79]; transcript levels of TMLHE, ALDH9A1, BBOX1, and OCTN2 were 10-, 6-, 1.8-, and 13-fold, respectively, higher in the liver of dairy cows at 1 wk postpartum than at 3 wk prepartum. In addition, concentration of carnitine in the liver was increased from 3 wk prepartum to 1 wk postpartum. In contrast, from 1 wk to 5 and 14 wk postpartum transcript levels of TMLHE, ALDH9A1, BBOX1, and OCTN2 and hepatic carnitine concentrations were declining [79]. Thus, it is likely that the observed changes in the expression of these genes account for the alterations of hepatic carnitine concentration during the transition period and the lactation cycle. Noteworthy, we also found that plasma concentrations of free fatty acids and hepatic carnitine concentrations at 1 wk, 5 wk, and 14 wk postpartum were positively correlated. Although it remains to be established that the bovine genes encoding TMLHE, ALDH9A1, BBOX1, and OCTN2 are direct PPAR*α* target genes, the positive correlations between plasma free fatty acids, which are endogenous activators of PPAR*α*, and hepatic carnitine concentrations during lactation are supportive for a role of PPAR*α* in the regulation of carnitine homeostasis in cattle. Besides these data from pregnant and lactating cows which provide indirect evidence for a PPAR*α*-dependency of carnitine homeostasis in cattle, unpublished data from our own group from cell culture experiments provide stronger evidence for a role for PPAR*α* in regulating genes involved in carnitine homeostasis in cattle. We found that treatment of bovine kidney cells with a PPAR*α* agonist increases transcript and protein levels of OCTN2. Whether the bovine BBOX1 gene is also regulated by PPAR*α* cannot be answered with certainty because BBOX1 is not expressed in this bovine kidney cell line (unpublished observation).

### 4.5. Chicken

Like in mammals, PPAR*α* has been shown to be highly expressed in chicken liver and to play an important role for the homeostasis of energy and lipid metabolism during fasting [80]. In addition, a high homology of avian PPAR*α* with mouse, rat, and human PPAR*α* [81, 82] and a similar expression pattern of PPAR*α* in tissues between chicken and rodents as well as humans has been reported [81, 82]. Moreover, a recent study demonstrated that PPAR*α* in the liver of laying hens can be strongly activated by the administration of clofibrate as evidenced from elevated transcript levels of classical PPAR*α* target genes [83]. In order to study the regulation of carnitine homeostasis by PPAR*α* in laying hens, we have recently performed a study with laying hens which were fed diets supplemented without (control) or with clofibrate [84]. Interestingly, this study revealed that treatment with clofibrate increased carnitine concentration not only in the liver but also in the whole egg, yolk, and albumen. On the molecular level, activation of PPAR*α* in the liver of clofibrate-treated hens could be demonstrated by elevated transcript levels of classical PPAR*α* target genes. In addition, this study demonstrated that OCTN2 but not genes encoding enzymes of carnitine biosynthesis in the liver are upregulated by clofibrate in the liver of laying hens [84], which indicates that increased carnitine concentrations in the liver of hens treated with clofibrate might be due to stimulation of OCTN2-mediated carnitine uptake from plasma into liver cells. Thus, the findings from this study suggested that PPAR*α* has an essential role in the regulation of carnitine homeostasis in hens like in mammalian species. Unlike in mice and pigs, however, PPAR*α* in laying hens appears to play a role only for regulating OCTN2-mediated carnitine uptake but not carnitine biosynthesis. In a further study, it has been investigated whether carnitine homeostasis in laying hens can be also influenced by the administration of nutritive PPAR*α* activators [85]. This study however failed to demonstrate an influence of either fish oil or conjugated linoleic acid (CLA) on carnitine homeostasis in laying hens. The lack of effect of nutritive PPAR*α* agonists on carnitine homeostasis, however, is not a contradiction to the abovementioned study but rather reflects the fact that activation of PPAR*α* by both fish oil and CLA in this study was negligible, which itself is likely due to the low binding affinity of n-3 PUFA and CLA isomers when compared to the synthetic PPAR*α* activator clofibrate.

### 4.6. Human

In contrast to extensive research on the regulation of carnitine homeostasis by PPAR*α* in animals, only few studies with limited significance are available to evaluate whether PPAR*α* regulates carnitine homeostasis in humans as well. One important reason for the limited significance of human studies is that, with few exceptions, most of them used plasma samples only, which is not appropriate for evaluating changes in carnitine homeostasis. To our knowledge only one study is available in the literature analyzing the change in the urinary profile of carnitine and its derivates in healthy adults in response to starvation [86], which is the physiological state of PPAR*α* activation. According to this study, 48 h starvation caused a slight decrease in the urinary excretion of free carnitine and a marked increase in that of acetyl carnitine. Albeit being speculative, the reduced urinary excretion of free carnitine in the starved subjects may be indicative of a PPAR*α*-induced increase in the tubular reabsorption of carnitine in the kidney which is possibly mediated by an upregulation of OCTN2. In another study with human subjects, from which skeletal muscle biopsies were taken, no change in skeletal muscle carnitine levels were found in patients under starvation conditions [87]. This finding however does not argue against the hypothesis that PPAR*α* is a regulator of carnitine homeostasis also in humans because the carnitine concentration in skeletal muscle, which is the main storage site for carnitine in the body, is expected to change only slightly even if OCTN2 is upregulated by PPAR*α* activation. Supportive of this assumption is the observation that concentrations of total carnitine in skeletal muscle also did not change in rats and pigs which were starved for 24 h [48, 67]. Further indications with regard to the regulation of carnitine homeostasis by PPAR*α* in humans may be expected to be obtained from clinical studies dealing with pharmacological PPAR*α* agonists (i.e., fibrates). However, according to our literature research no clinical studies investigating the efficacy of different fibrates (gemfibrozil, bezafibrate, fenofibrate, etiofibrate, ciprofibrate) for blood lipid modifying purposes were found that also reported on either plasma or urinary carnitine levels.

## 5. Evidence for a Role of Other PPAR Isotypes in Regulating Genes Involved in Carnitine Homeostasis

Besides PPAR*α*, two other PPAR isotypes, PPAR*γ*, which is expressed in two different full-length translated isoforms (PPAR*γ*1, PPAR*γ*2), and PPAR*δ*, exist in mammals and birds. The distribution pattern and expression levels of the PPARs show great differences between tissues. Whereas PPAR*α* is highly expressed in tissues with high rates of fatty acid oxidation (liver, kidney, myocardium, skeletal muscle), PPAR*γ*1 is poorly expressed in these tissues. Both PPAR*α* and PPAR*γ*1 are found in cells of the immune system and the vessel wall and in epithelial cells. The adipocyte-specific PPAR*γ*2 isoform is exclusively and highly expressed in adipose tissue. PPAR*δ* is ubiquitously expressed and the predominant PPAR isotype in skeletal muscle. To our knowledge only one study has been published investigating the role of other PPAR isotypes than PPAR*α* on genes involved in either carnitine uptake or carnitine biosynthesis [88]. According to this study the expression of OCTN2 in the colon is upregulated by PPAR*γ* in humans and mice and thereby contributes to local and systemic carnitine homeostasis. Whether PPAR*γ* is also a transcriptional regulator of genes encoding enzymes of the carnitine biosynthesis pathway has not been investigated in this study. In addition, the role of PPAR*δ* in regulating genes involved in carnitine homeostasis has not been addressed so far. However, PPAR*δ* has similar and partially overlapping functions as PPAR*α*, in particular with regard to fatty acid catabolism [89]. For instance, genes encoding proteins of the carnitine shuttle system, such as carnitine-palmitoyltransferase I [90] and carnitine-acylcanitine translocase [91], were shown to be regulated by both PPAR*α* and PPAR*δ*. Thus, it would be not unlikely that PPAR*δ* is also a transcriptional regulator of OCTN2 and genes of the carnitine biosynthesis pathway. This, however, remains to be shown in future studies.

## 6. Conclusions

**Figure 1:**
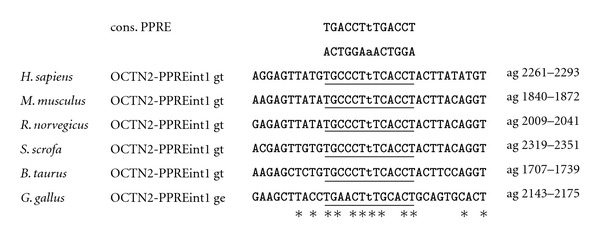
Sequence alignment of the functional PPRE in the intron 1 of human, mouse, rat, pig, cattle, and chicken OCTN2. The PPRE, which is comprised of two hexanucleotides separated by a single nucleotide, termed direct repeat 1, is underlined. Matching nucleotides are shown by asterisks. Chromosomal localization, accession number of cDNA, and genomic DNA sequences from Genbank of NCBI are: hOCTN2 chr.5, AF057164 cDNA, AC118464 genomic DNA; mOCTN2 chr.11, BC031118 cDNA, AL596182 genomic DNA; rOCTN2 chr.10, NM_019269 cDNA, AC120085 genomic DNA; sOCTN2 chr.2, AK393575/AK394838/FS677719 cDNA, CU372899 genomic DNA; cOCTN2 chr.7, NM_001046502 cDNA, AC149665 genomic DNA; chOCTN2 chr.13, NM_001045828 cDNA, JH374679 genomic DNA.

**Figure 2:**
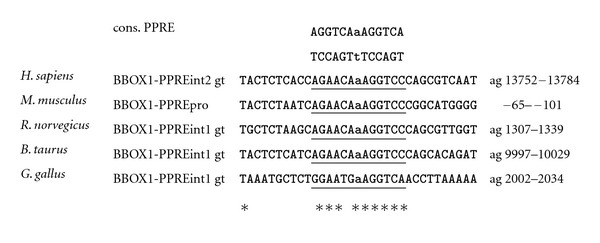
Sequence alignment of the functional PPRE in the promoters, intron 1 and intron 2, respectively, of human, mouse, rat, pig, cattle, and chicken BBOX1. The PPRE, which is comprised of two hexanucleotides separated by a single nucleotide, termed direct repeat 1, is underlined. Matching nucleotides are shown by asterisks. The BBOX1-PPRE for *S. scrofa* is not shown due to gaps in the first and second intron. Chromosomal localization, accession number of cDNA, and genomic DNA sequences from Genbank of NCBI are: hBBOX1 chr.11, NM_003986 cDNA, AC015756 genomic DNA; mBBOX1 chr.2, NM_130452 cDNA, AL691416 genomic DNA; rBBOX1 chr.3, NM_022629/FQ210746 cDNA, AABR03024937 genomic DNA; cBBOX1 chr.15, NM_001101881 cDNA, genomic DNA; sBBOX1 chr.2, AK393528/AK391112 cDNA. CU694591 genomic DNA; chBBOX1 Chr.5, BX936048 cDNA, JH374511 genomic DNA.

**Figure 3:**
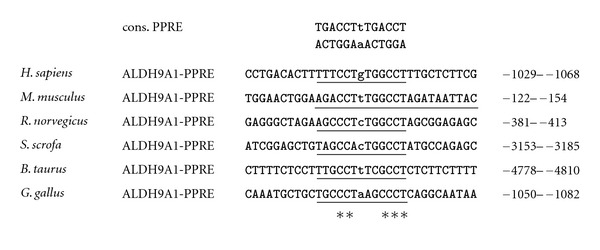
Sequence alignment of the functional PPRE in the promoter of human, mouse, rat, pig, cattle, and chicken ALDH9A1. The PPRE, which is comprised of two hexanucleotides separated by a single nucleotide, termed direct repeat 1, is underlined. Matching nucleotides are shown by asterisks. Chromosomal localization, accession number of cDNA, and genomic DNA sequences from Genbank of NCBI are: hALDH9A1 chr.1, AK392520 cDNA, AL451074, genomic DNA; mALDH9A1 chr.1, NM_019993, cDNA, AC113970 genomic DNA; rALDH9A1 chr.13, NM_022273 cDNA, AABR06075994 genomic DNA; sALDH9A1 chr.4, AK392520 cDNA, CU468388 genomic DNA; cALDH9A1 chr.3, BC105335 cDNA, AAFC03093575 genomic DNA; chALDH9A1 Chr.8, BU460904 cDNA, JH374592 genomic DNA.

Comparison of data from genetic and animal studies with mice, rats, pigs, cows, and laying hens and from human studies on the regulation of genes involved in carnitine homeostasis by PPAR*α* suggests that carnitine homeostasis, which is intrinsically linked with lipid catabolism, is well conserved across different species. This confirms recent observations from genome-wide comparative analysis of gene regulation by PPAR*α* between mouse and human demonstrating that at least the role of PPAR*α* as a master regulator of hepatic lipid catabolism is well conserved [20]. However, despite demonstrating a well conserved role of PPAR*α* as a key regulator of carnitine homeostasis in general, our comprehensive analysis shows that this assumption particularly applies to the regulation of genes involved in carnitine uptake (OCTN2) by PPAR*α* which is obviously highly conserved across species. The highly conserved regulation of OCTN2 by PPAR*α* is possibly explained by the fact that the sequence of the functional PPRE identified in the mouse OCTN2 gene is completely identical (100%) between mouse, rat, pig, cattle, and even human (Figure 1). The comparison of studies in pigs with studies in mice and rats, however, shows that the upregulation of OCTN2 in the liver by PPAR*α* activation is clearly stronger in rodents than in pigs, which is in line with the view that nonproliferating species (pig, human, nonhuman primates) generally show a weaker response to PPAR*α* activation than proliferating species (mice, rats). In contrast, regulation of genes involved in carnitine biosynthesis (BBOX1, ALDH9A1) by PPAR*α* appears less well conserved across species, which is demonstrated by the fact that PPAR*α* activation causes upregulation of genes involved in carnitine biosynthesis in mice, pigs and cattle but not in rats and chicken. The reasons underlying these species specificities cannot be simply explained by differences in the PPAR*α* expression level between species because mice and rats, for instance, exhibit comparably high hepatic PPAR*α* expression levels. In the case of BBOX1 differences in the nucleotide sequence of the functional PPRE of the BBOX1 gene between mouse and rat also cannot explain this species specificity because this PPRE shares a complete (100%) sequence identity between mouse and rat and even human and cattle (Figure 2). One factor that may account for the species specificity regarding BBOX1 regulation by PPAR*α* is the different location of the translation start site of the BBOX1 gene between mouse (translation start site in the first exon) and rat (translation start site in the second exon). In addition, a species specific expression pattern of transcriptional coregulators in the liver may be causative for the different regulation of BBOX1 by PPAR*α* between mouse and rat. By contrast, a small discrepancy in the sequence of the functional PPRE of the ALDH9A1 promoter between mouse and rat (one nucleotide in the proximal half site of the PPRE is different) could explain the species specificity regarding ALDH9A1 regulation by PPAR*α* (Figure 3). Nevertheless, multiple factors may be responsible for the different regulation of carnitine biosynthesis by PPAR*α* across different species and, therefore, further research is required to unravel the underlying reasons. Overall, our comparative analysis indicates that PPAR*α* is not only a master transcriptional regulator of fatty acid catabolism, ketogenesis, and gluconeogenesis but also of carnitine homeostasis—a role which is well conserved across species.
